# The Effect of Educational Intervention on Knowledge, Attitude, and Practice of Women towards Breast Cancer Screening

**DOI:** 10.1155/2022/5697739

**Published:** 2022-05-26

**Authors:** Tayebeh Rakhshani, Maryam Dada, Seyyed Mansour Kashfi, Amirhossein Kamyab, Ali Khani Jeihooni

**Affiliations:** ^1^Nutrition Research Center, Department of Public Health, School of Health, Shiraz University of Medical Sciences, Shiraz, Iran; ^2^Department of Public Health, School of Health, Shiraz University of Medical Sciences, Shiraz, Iran; ^3^Department of Community Medicine, School of Medicine, Fasa University of Medical Sciences, Fasa, Iran

## Abstract

**Background:**

Early identification of breast cancer may result in earlier treatment and a lower mortality rate. This fact has resulted in the development of screening programs to detect breast cancer in its early stages; thus, the current study sought to investigate the influence of educational intervention on knowledge, attitude, and practice about breast cancer screening in Izeh, Khozestan Province, Iran.

**Materials and Methods:**

This experiment was carried out on the women of Izeh city in 2019. This study included 120 women who were randomized into the experimental and control groups at random (60 in the experimental and 60 in the control groups). Before and two months after the intervention, data were collected using a researcher-created questionnaire by the control and education groups. The intervention program for the intervention group included eight educational sessions over the course of two months. The SPSS 20 statistical program was used to examine the data, as well as the paired *t*-test, independent *t*-test, and Chi-square.

**Results:**

There was no significant difference between the experimental and control groups' mean scores of knowledge, attitude, and practice prior to the educational intervention, but a significant difference was observed after the intervention, with the experimental group obtaining significantly higher mean scores of knowledge, attitude, and practice (*P* = 0.05).

**Conclusion:**

In the present study, the educational intervention on the knowledge, attitude, and practice towards breast cancer screening led to the increased scores of the experimental group compared to control group.

## 1. Introduction

The World Health Organization reported 7.6 million deaths from cancer in 2005 [1]. Breast cancer is the second most frequent malignancy in women, after skin cancer. It is also the second most frequent cancer of all forms and the leading cause of death in both developed and developing countries for women [1].

According to global statistics, 80000 persons per 100000 are afflicted with the disease [2], and 519000 women die from breast cancer each year, with more than 70% occurring in low-income nations [3].

According to the most recent estimates, 1383500 persons were diagnosed with breast cancer in 2010, with 458400 dying as a result of the condition. Thus, in past years, breast cancer was the most frequent malignancy among women and the second largest cause of death. However, with 458400 fatalities per year, cancer presently ranks first in cancer mortality among women worldwide, with lung cancer coming in second with 427400 deaths per year [4].

The disease rate has been rising in Iran in recent years, and it has been the most prevalent cancer among Iranian women since 1998, with the annual rate increasing by 2% between 2002 and 2007 [3]. The average age of development of breast cancer in Iran is estimated to be 48.8, with the highest incidence of malignancy observed in the age group of 40-49 years [4].

The risk factors for this cancer include family history, age of first pregnancy, early onset of menstruation, late menopause, obesity after menopause, alcohol consumption, smoking, physical inactivity, body mass index, hormone therapy, breast density, and exposure to chest radiography. However, job stress, women's night work shifts, and lifestyle are definitely influential [5–8].

Despite the reduction in breast cancer mortality, especially in developed countries, the disease is still a major challenge for health policy makers in developing countries, including Iran [9, 10]. Hence, early detection of cancer is critical. It is estimated that more than a third of cancers are preventable [11], and breast cancer is one of the few cancers that can be diagnosed early [12]. Early detection of breast cancer may lead to early treatment and reduced mortality. This fact has led to the emergence of screening programs to detect breast cancer in the early stages in which treatment has the greatest impact on clinical outcomes [7].

About half of the patients with early diagnosis of breast cancer spend the rest of their lives without recurrence, and a third die from the disease. Thus, it is obvious that breast cancer prevention and early diagnosis are among the vital factors in controlling the disease and increasing the patients' survival [13].

Screening methods can reduce mortality by over 25%. Prompt diagnosis has a great impact on the treatment process and the patients' survival as well. Experts believe that if cancer is diagnosed at an early stage, the survival of the patients in the first 5 years will be 97%. They suggest that women undergo regular and monthly screening methods [14]. There are three screening methods for early detection of breast cancer including mammography, clinical breast examination, and breast self-examination, in the order of importance [15, 16]. Breast self-examination is a screening method that does not require specialized equipment and staff due to its simplicity, cost-effectiveness, and efficiency and is performed by the person herself after being trained. If the person performs breast self-examination regularly and accurately, she can detect even smaller-than-1 cm glands [17]. According to the findings of several research, the world as a whole has a low rate of regular and monthly breast self-examination. Although 82% and 61% of women in Saudi Arabia were aware of the benefits of breast self-examination and mammography, respectively, only 41.2 percent and 18.2 percent had performed breast self-examination and nondiagnostic mammography, respectively [18]. Despite the benefits of breast self-examination, few women do it and some do not even know how to do it. There is evidence that women trained by BSE physicians or nurses are more likely to do breast self-examination [19]. Given that breast cancer can be detected by the individual through breast self-examination, informing women about it and teaching them how to do breast examination properly seem necessary [19]. Studies show that one of the most important factors in promoting breast cancer screening awareness and practices is education [20]. In their study, Taha et al. reported that the mean scores of cancer awareness, breast self-examination, risk factors, and symptoms were 2.10 ± 0.67, 34.10 ± 0.77, 8.05 ± 3.1, and 6.80 ± 2.73, respectively. Their results indicated a low level of women's awareness of screening [21].

Women make up half of the population of any society and meanwhile nurture all members of the society. In the current Iranian health system, women with health records in health centers can be examined for free, but due to the lack of regular visits and the low level of their knowledge and attitude towards breast cancer, this program has faced problems. On the other hand, since there is no systematic program to teach and learn breast cancer screening methods in the country, even in urban communities, and as breast cancer is the most common cancer among women and a major problem in health priorities at the national and regional levels, the present study entitled the effect of in-person education on knowledge, attitude, and practice towards breast cancer screening was conducted in Izeh city in 2019.

## 2. Materials and Methods

### 2.1. Study Design and Participants

This experimental study was carried out in 2019 in Izeh city, Khozestan Province, Iran. The study population consisted of the women who referred to Izeh community health centers in 2019.

#### 2.1.1. Inclusion and Exclusion Criteria

The inclusion criteria were as follows: having a health record in a health center, not having a special disease (such as a history of cancer and depression, cardiovascular disease), and not having cancer. The exclusion criteria were unwillingness to participate at any time of the study, more than two absences from the training sessions, and changing the dwelling place.

### 2.2. Sample Size

The sample size was estimated to be 47 for each group, using the comparison of two means and taking into account the dropout rate of 20% as well as the alpha of 0.05 and 80% study power. However, 60 people per group were examined in the final analysis due to the sample dropout [22]. The 120 participants were divided into two groups using the semirandom sampling method (60 individuals in the intervention group and 60 in the control group).

(1) $$\begin{array}{c} n=\frac{{\left(Z_{1-\left(\alpha /2\right)}+Z_{1-\beta }\right)}^{2}\left(3.2^{2}+3.37^{2}\right)}{{\left(18.1-16.2\right)}^{2}}. \end{array}$$

### 2.3. Sampling Method

In this investigation, the cluster sampling method was used. Izeh city had 5 comprehensive health centers and 12 attached and unattached health homes and centers; each health home and center was designated a cluster.

Then, two health centers and two health homes were chosen at random, with one center and one health home serving as the experimental group and one center and one health home serving as the control group.

Then, among the selected health homes and centers, 60 women were randomly chosen from one health home and one health center for the experimental group; then, 60 women were also chosen from the remaining health home and health center serving as the control group of the study.

### 2.4. The Data Collection Tools

To gather information for this study, the researchers used a questionnaire to gather demographic information about the women (such as their occupation, education, and economic status), as well as a researcher-made questionnaire to elicit information about the women's breastfeeding history and any underlying diseases (such as diabetes, cardiovascular disease, and another cancer).

### 2.5. Knowledge, Attitude, and Practice Questionnaire

On the knowledge level, there were 18 questions (related to breast cancer screening, including the information about its necessity, as well as the time of onset, frequency, location, and ways of doing breast self-examination in each cycle, and the time of onset and frequency of clinical examinations and mammography). The Likert scale was used to score the responses (1 = severely disagree, 2 = disagree, 3 = no idea, 4 = agree, and 5 = highly agree). The lowest and highest scores obtained were 18 and 90, respectively.

There were 20 attitude questions, all of which were about screening for early detection and treatment of cancer and its implications. The Likert scale was used for scoring (1 = severely disagree, 2 = disagree, 3 = no idea, 4 = agree, and 5 = highly agree). The lowest and highest scores were 20 and 100, respectively.

There were seven practice-related questions, separated into three categories: breast self-examination, clinical breast examination, and mammography. The Likert scale was used to score the responses (1 = severely disagree, 2 = disagree, 3 = no idea, 4 = agree, and 5 = highly agree). The lowest and highest possible scores were 7 and 35, respectively. The questionnaire's reliability and validity were assessed in this study, and its Cronbach's alpha was 0.82 percent (Supplementary Files-Research Project Questionnaire).

### 2.6. Educational Intervention Program

In the experimental group, knowledge, attitude, and practice were the foci of the educational intervention. It consisted of eight 60-minute sessions held weekly over the course of two months for the experimental group. The details of the training sessions are presented in Table 1. The Breast Cancer Screening Guide published by the Ministry of Health was used to provide educational information in the training sessions [9].

In the control group, no intervention was performed.

### 2.7. Statistical Analysis

The SPSS 20 software was used to analyze the data, and the normality of the data was first determined using the Kolmogorov-Smirnov test. The data was also described using frequency, mean, and standard deviation indices, and data was analyzed using the paired *t*-test, independent *t*-test, and Chi-square test. In all tests, the significance threshold was set at 0.05.

### 2.8. Ethics Approval Code

This study was approved by the ethics committee of Shiraz University of Medical Sciences as the project number (IR.SUMS.REC. 97-01-04-19005).

### 2.9. Results

The demographic and background information of the participants is shown in Table 2. The mean and standard deviation of the age of the participants in the intervention and control groups were 33.65 ± 8.56 and 33.65 ± 8.25, respectively; the independent *t*-test showed no significant difference between the experimental and control groups in terms of age (*P* = 0.22).

The Chi-square test indicated that there was no significant difference between the experimental and control groups in terms of education (*P* = 0.56), economic status (*P* = 0.07), occupation (*P* = 0.24), number of deliveries (*P* = 0.22), type of previous delivery (*P* = 0.22), history of breastfeeding (*P* = 0.22), and underlying disease (*P* = 0.27) and history of breast self-examination (*P* = 0.32).

The independent *t*-test showed that prior to the educational intervention, there was no significant difference between the two groups in terms of their mean scores of knowledge, attitude, and practice, but the result completely reversed after the intervention, and the experimental group obtained higher mean scores of knowledge, attitude, and practice. The difference was statistically significant (*P* < 0.05) (Table 3).

The results of the paired *t*-test indicated a significant difference in the experimental group in terms of the mean scores of knowledge, attitude, and practice before and after the intervention (*P* < 0.05) (Table 4).

According to the results of the paired *t*-test, no significant difference was observed in the control group in terms of the mean scores of knowledge, attitude, and practice before and after the intervention (*P* > 0.05) (Table 5).

## 3. Discussion

In the present study, educational intervention led to an increase in the mean knowledge of the experimental group compared to that of the control group. This finding is consistent with the results of the studies conducted by Awwad et al., Noman et al., Ibitoye et al., and Dadsetan et al. and on learning knowledge from longitudinal data of mammograms to improving breast cancer risk prediction [22–25]. It is consonant with another study by Mohsenipouya et al., who aimed at investigating the use of educational intervention in breast cancer screening in northern Iran [26]. Also, it was consistent with the studies by Heidari et al., Sabeg et al., and Sadeghi et al. [27–29].

The finding of this study was also in line with those of the studies by Noman et al. on effect of educational intervention of screening practices on the knowledge and attitude of primary school teachers in Malaysia [30], and Alomair et al. on the effect of educational intervention on screening practices of the Saudi women [31].

The possibility of reason of increase acknowledges was the educational program, because the educational program was necessary to increase knowledge and create proper health attitudes and beliefs and to perform breast self-examination accurately and correctly. However, acquiring necessary skills was also essential. On the other hand, the high mean score of knowledge in our study could be due to the fact that in general, in Iran and other developing countries, women's increased information and desire for acquiring knowledge have led to some practice for early diagnosis of breast cancer, and, as a result, they are more aware of this disease than before.

In the present study, the educational intervention led to an increase in the mean attitude of the experimental group compared to the control group. Possibly, the reason for the increase in attitude was the training program which justifies the result, that is to say, the level of knowledge directly affected the formation of correct attitudes in women. In the present study, women's high levels of knowledge changed their attitudes, and that, in turn, had a positive effect on breast self-examination, consistent with the results of the studies conducted by Sadoh et al., Samami et al., and Maheri et al. [32–34], and also in line with another study by Alsaraireh and Darawad on the impact of breast cancer educational intervention on the women's knowledge, attitude, and performance in Jordan [35]. However, the findings of our study are not consistent with those of the study by Khani Jeihooni et al. who examined the effect of educational intervention on breast cancer screening in Iranian women [36]. They found out that the mean score of attitude increased in the experimental group after the intervention, but it was not statistically significant [36]. Attitude is one of the issues that requires a long time to change and is rooted in the person's beliefs and understanding. To change attitudes, the person's root beliefs must be identified and corrected.

The possible positive effect of educational program in the experimental group is also consistent with the results of the studies conducted by Dieli-Conwright et al., Abo Al-Shiekh et al., and Prusty et al. [37–39].

Also, the results of the present study are consistent with the results of the study by Godfrey et al. who assessed breast cancer knowledge and breast self-examination on 204 female students of Kampala, Uganda [40]. However, it was not consistent with the results of the study by Sargazi et al. on the effect of educational intervention on early breast cancer screening in Zahedan [41].

According to the results, the mean scores of knowledge in the experimental group before and after the educational intervention, respectively, showing a significant increase. That is, knowledge-focused interventions were effective on screening. This is consistent with the results of a study by Tuna et al. entitled Online Education in Teaching Breast Self-Examination [42]. In their study, Tuna et al. examined 1679 women and reported that the mean scores of the participants' knowledge of breast self-examination was 46.5 (14%) before the education, 77.4 (11%) one month after the education, and 76.7 (9.52%) six months later.

The results of the present study showed that the mean scores of attitude in the experimental group were 64.08 ± 7.31 before the educational intervention and 79.53 ± 4.47 after it, showing a significant increase. In other words, attitude-focused interventions could lead to increased attitudes toward breast cancer screening. This finding is in line with the results of the study by Ghasemi and Kheivani (2014). They randomly examined 50 women working in universities and reported that the mean attitude score had changed from 74.5 ± 14.7 to 82.2 ± 10.2 [43].

According to the results of this study, the mean score of practice in the experimental group significantly increased after the educational intervention (27.86 ± 4.03) compared to the preintervention score (15.46 ± 4.14). In other words, practice-focused interventions could lead to an increase in screening practices, consistent with the results of the study by Tuna et al. entitled Online Education in Teaching Breast Self-Examination [42]. They examined 1679 women and reported that the rate of systematic breast self-examination among the women increased from 30.8% to 47.8% after the education program, which was significantly different [42].

The results of the present study indicated that before the educational intervention, the mean score of knowledge in the control group (59.21 ± 7.06) was higher than in the experimental group (53.51 ± 6.85), and the mean score of practice was also higher in the control (20.66 ± 4.50) than the experiment group (15.46 ± 4.14). This could be partly due to the difference between the two groups in terms of their education levels, as the control group had higher education than the other group, and the results of many studies have shown that higher education would lead to increased knowledge and practice towards breast cancer screening. In general, people with university education are more likely to undergo cancer screening than those with lower levels of education.

In the end, it can be said that breast cancer is one of the diseases, of which the major part of prevention is left to individuals, and people's knowledge, attitude, and function to prevent and control the disease, especially through screening, attending educational sessions, and doing breast self-examination are very important. Thus, conducting studies on different age groups with the aim of increasing knowledge of cancer screening is an important step towards preventing and controlling the disease.

One of the weaknesses of the present research is the short-term follow-up of the implemented educational program.

## 4. Conclusion

In the present study, the educational intervention on the knowledge, attitude, and practice towards breast cancer screening led to the increased scores of the experimental group compared to the control one. Therefore, it is suggested that in the implementation of cancer prevention programs, especially breast cancer, serious efforts be made to educate and inform women in the community in order to increase their knowledge and change their attitudes. Without multiple intervention strategies and especially paying attention to health education, programs cannot be successful because health education is the major component of health promotion.

## Figures and Tables

**Table 1:** The educational intervention program was as follows.

Session	Objective	Subject	Time duration (minute)	Training method	Educator
1^st^	The aim of educational sessions	Acquainting the group members with each other and with the psychologist expressing objectives Familiarity with different types of cancers and breast cancer	60	Lectures as well as Q&A	Expert group and midwifery expert
2^nd^	Screening	Training on breast cancer screening types of screening and advantages and disadvantages of each	60	Lectures, group discussions, and Q&A	Psychiatrist and midwifery expert
3^rd^ & 4^th^	Knowledge, attitude, and practice	Definition of knowledge, attitude, and practice—assessing the levels of knowledge, attitude and practice of the participants	60	Educational videos and posters	Consulting expert and midwifery expert
5^th^ & 6^th^	Role of knowledge, attitude, and practice in screening	Determining the advantages and benefits of screening in early cancer diagnosis	60	Lectures, group discussions, and Q&A	Expert group and midwifery expert
7^th^ & 8^th^		Review of previous contents and summary and final evaluation	60	Lectures, group discussions and Q&A	Midwifery expert

**Table 2:** Comparison of frequency distribution of primary variables of the participants in two groups.

Variable		Control group (%)	Experimental group (%)	*P* value
Education	Under diploma	11 (18.3)	9 (15)	0.56
	Diploma	29 (48.4)	31 (51.7)	
	University degree	20 (33.3)	20 (33.3)	

Economic status	1-2 million	14 (23.3)	6 (10)	0.07
	2-4 million	32 (53.3)	43 (71.7)	
	More than 4 million	14 (23.3)	11 (18.3)	

Occupation	Housewife	53 (88.3)	57 (95)	0.24
	Employee	7 (11.7)	3 (5)	

Number of deliveries	No delivery	14 (23.3)	8 (13.3)	0.27
	One delivery	8 (13.3)	13 (21.7)	
	More than one delivery	38 (36.6)	39 (65)	

Type of previous delivery	No history of delivery	14 (23.3)	8 (13.3)	0.31
	Natural delivery	30 (50)	31 (51.7)	
	Cesarean section	16 (26.7)	21 (35)	

History of breastfeeding	Yes	48 (80)	52 (86.7)	0.31
	No	12 (20)	8 (13.3)	

Underlying disease	Yes	12 (20)	5 (8.3)	0.1
	No	48 (80)	55 (91.7)	

Has a history of breast self-examination	Yes	14 (23.3)	3 (5)	0.32
	No	46 (76.7)	57 (95)	

^∗^Chi-square test.

**Table 3:** Comparison of mean knowledge, attitude, and practice scores of the two groups before and after the intervention.

*P* value^∗^	Preintervention		*P* value^∗^	Postintervention		Variable
	Control	Experimental		Control	Experimental	
	Mean ± SD	Mean ± SD		Mean ± SD	Mean ± SD	
0.001	59.78 ± 6.13	83.48 ± 4.47	0.12	59.21 ± 7.06	53.51 ± 6.85	Knowledge
0.001	64.13 ± 5.64	79.53 ± 4.47	0.26	65.46 ± 6.18	64.8 ± 7.31	Attitude
0.001	20.93 ± 3.01	27.86 ± 4.03	0.31	20.66 ± 4.50	15.46 ± 4.14	Practice

^∗^Independent *t*-test.

**Table 4:** Comparison of mean knowledge, attitude, and practice in the experimental group before and after the intervention.

*P* value^∗^	Postintervention	Preintervention	Variable
	Mean ± SD	Mean ± SD	
0.001	83.48 ± 4.47	53.51 ± 6.85	Knowledge
0.001	79.53 ± 4.47	64.8 ± 7.31	Attitude
0.001	27.86 ± 4.03	15.46 ± 4.14	Practice

^∗^Paired *t*-test.

**Table 5:** Comparison of mean knowledge, attitude, and practice before and after the intervention in the control group.

*P* value^∗^	Postintervention	Preintervention	Variable
	Mean ± SD	Mean ± SD	
0.50	59.78 ± 6.13	59.21 ± 7.06	Knowledge
0.17	64.13 ± 5.64	65.46 ± 6.18	Attitude
0.70	20.93 ± 3.01	20.66 ± 4.50	Practice

## Data Availability

The data used to support the findings of this study will be provided on request.

## Supplementary Materials

Supplementary Materials Research project questionnaire.
