# Supplementary material for: The Effect of Educational Intervention on Knowledge, Attitude, and Practice of Women towards Breast Cancer Screening
Source: Int J Breast Cancer. 2022 May 26;2022:5697739. doi: 10.1155/2022/5697739 (PMC9162849; doi:10.1155/2022/5697739)
Supplement: Supplementary Materials — Research project questionnaire. [file 5697739.f1.docx]

**Research Project Questionnaire**

Dear Madam,

Thank you for your time, the present study examines the effect of breast self-examination training on women who referred to Izeh city community health centers; therefore, please read and answer the questions related to this questionnaire carefully.

The information obtained from this questionnaire will be kept by the researcher. Thank you for your cooperation.

**Personal details**

1. Age by year …
2. Marital status: Single □ Married □ Divorced □ Widowed □
3. Level of education: Elementary □ Secondary □ High school □ Tertiary education □
4. Economic situation: Weak □ Moderate □ Good □
5. Occupation: Employee □ Housewife □
6. Insurance status: I am covered by insurance □ I am not covered by insurance □
7. Number of deliveries:
8. Type of delivery:
9. Age of first menstruation:
10. Age of first marriage:
11. Underlying disease
12. Breastfeeding history
13. How did you get information about health and disease mostly?

Asking the staff and personnel of health centers

Asking office doctors, mass media, social media and the internet

Other …

Mark the correct answer for each of the following questions:

1. Do you have a history of uterine or breast cancer? Yes □ No □
2. Do you have a positive family history of uterine or breast cancer? Yes □ No □
3. Breast self-examination training history? Yes □ No □
4. History of breast pain? Yes □ No □
5. Positive family history of breast cancer? Yes □ No □
6. Breastfeeding history? Yes □ No □

**Knowledge**

Please select the best option for the multiple choice questions in this section:

1. A woman should start breast self-examination at the age of 20

Strongly agree □ Agree □ No comment □ Disagree □ Strongly disagree □

1. Breast self-examination should be performed monthly

Strongly agree □ Agree □ No comment □ Disagree □ Strongly disagree □

1. The correct time to perform a breast self-examination is 7-9 days after the first day of menstruation

Strongly agree □ Agree □ No comment □ Disagree □ Strongly disagree □

1. After menopause: Breast self-examination should be done on a regular day of each month

Strongly agree □ Agree □ No comment □ Disagree □ Strongly disagree □

1. The correct technique for performing a breast self-examination is a circular motion with finger pads

Strongly agree □ Agree □ No comment □ Disagree □ Strongly disagree □

1. The initial purpose of breast self-examination is to become familiar with breast tissue

Strongly agree □ Agree □ No comment □ Disagree □ Strongly disagree □

1. Does a breast mass which is 0.6 cm in size and is relatively firm and stable in the palpation need specialized follow-up?

Strongly agree □ Agree □ No comment □ Disagree □ Strongly disagree □

1. It is abnormal to have secretion from one nipple when it is spontaneously and without pressure

Strongly agree □ Agree □ No comment □ Disagree □ Strongly disagree □

1. Two weeks before ovulation, the breasts may become sensitive during the menstrual cycle

Strongly agree □ Agree □ No comment □ Disagree □ Strongly disagree □

1. The correct method of breast self-examination includes inspection and palpation of both breasts

Strongly agree □ Agree □ No comment □ Disagree □ Strongly disagree □

1. During performing a breast self-examination, the best result is obtained when a combination of horizontal and circular movements are performed, and when a square pillow is placed under the shoulders

Strongly agree □ Agree □ No comment □ Disagree □ Strongly disagree □

1. The most important value of breast self-examination in women's health; is only the initial diagnostic method allowing a woman to check her breasts’ health by herself

Strongly agree □ Agree □ No comment □ Disagree □ Strongly disagree □

1. The time required to perform a self-examination of each breast is 7 minutes

Strongly agree □ Agree □ No comment □ Disagree □ Strongly disagree □

1. Breast cancer is not palpable or visible in the early stages

Strongly agree □ Agree □ No comment □ Disagree □ Strongly disagree □

1. Currently, the chance of developing breast cancer is 1 in 8 women

Strongly agree □ Agree □ No comment □ Disagree □ Strongly disagree □

1. Applicable methods for early detection of breast cancer are breast self-examination, clinical examination and mammography

Strongly agree □ Agree □ No comment □ Disagree □ Strongly disagree □

1. First menstruation before the age of 12 and old age Family history are potential risk factors of increased incidence of cancer

Strongly agree □ Agree □ No comment □ Disagree □ Strongly disagree □

1. There are two reasons why women do not embrace the accepted standards for breast self-examination. One is that women do not have enough knowledge and the second is that they are not completely supported

Strongly agree □ Agree □ No comment □ Disagree □ Strongly disagree □

**Attitude**

1. I'm too young to develop with breast cancer so I do not need to have a breast self-examination

Strongly agree □ Agree □ No comment □ Disagree □ Strongly disagree □

1. If breast cancer is treated in time, I will live a normal life

Strongly agree □ Agree □ No comment □ Disagree □ Strongly disagree □

1. If I find a painless mass in my breast, I worry it might be cancer

Strongly agree □ Agree □ No comment □ Disagree □ Strongly disagree □

1. Breast cancer is a serious illness

Strongly agree □ Agree □ No comment □ Disagree □ Strongly disagree □

1. Early detection using mammography reduces the complications of cancer

Strongly agree □ Agree □ No comment □ Disagree □ Strongly disagree □

1. Daily exercise can help prevent breast cancer

Strongly agree □ Agree □ No comment □ Disagree □ Strongly disagree □

1. Life will be hard for me if I develop with breast cancer

Strongly agree □ Agree □ No comment □ Disagree □ Strongly disagree □

1. Breast cancer can spread to other parts of the body

Strongly agree □ Agree □ No comment □ Disagree □ Strongly disagree □

1. In my opinion, breast cancer is a dangerous and fatal disease

Strongly agree □ Agree □ No comment □ Disagree □ Strongly disagree □

1. Developing breast cancer disrupts my marital and social relationships

Strongly agree □ Agree □ No comment □ Disagree □ Strongly disagree □

1. I perform breast self-examination because it is convenient and simple

Strongly agree □ Agree □ No comment □ Disagree □ Strongly disagree □

1. Taking a considerable amount of fruit and vegetables prevents breast cancer

Strongly agree □ Agree □ No comment □ Disagree □ Strongly disagree □

1. I will feel confident after having breast examination by a doctor

Strongly agree □ Agree □ No comment □ Disagree □ Strongly disagree □

1. I undergo mammography annually to find out about my condition

Strongly agree □ Agree □ No comment □ Disagree □ Strongly disagree □

1. Daily exercising can improve my body's ability in prevention of breast cancer

Strongly agree □ Agree □ No comment □ Disagree □ Strongly disagree □

1. Breast examination by a doctor is embarrassing for me

Strongly agree □ Agree □ No comment □ Disagree □ Strongly disagree □

1. The cost of mammography is high

Strongly agree □ Agree □ No comment □ Disagree □ Strongly disagree □

1. I do not perform breast self-examination due to the fear of having cancer

Strongly agree □ Agree □ No comment □ Disagree □ Strongly disagree □

1. I do not have the determination to exercise regularly

Strongly agree □ Agree □ No comment □ Disagree □ Strongly disagree □

1. Preparing vegetables is time consuming

Strongly agree □ Agree □ No comment □ Disagree □ Strongly disagree □

**Practice-related Questions**

**Breast Self-examination:**

1. I am able to perform a breast self-examination without the help of others

Strongly agree □ Agree □ No comment □ Disagree □ Strongly disagree □

1. I can afford to undergo regular breast examinations and mammograms

Strongly agree □ Agree □ No comment □ Disagree □ Strongly disagree □

1. I have the ability to do daily physical activities

Strongly agree □ Agree □ No comment □ Disagree □ Strongly disagree □

1. I have a regular schedule for annual mammograms

Strongly agree □ Agree □ No comment □ Disagree □ Strongly disagree □

1. I have a significant amount of fruits and vegetables in my daily routine

Strongly agree □ Agree □ No comment □ Disagree □ Strongly disagree □

1. In case of any problems, I gave access to an available center for consultation and examination

Strongly agree □ Agree □ No comment □ Disagree □ Strongly disagree □

What is your most informative source about breast cancer screening tests? (You may answer more than once)

- Mass media
- Books
- Newspapers
- Magazines
- Friends and acquaintances
- Doctor or health care staff
- Other

**Clinical Breast Examination:**

1. First step: Inspection was performed: correctly incompletely not performed

- Sitting position
- Arms by the side
- Breasts inspection looking for any change

1. Second step: Inspection

- Arms over head
- Pay attention to the upward movement of the breasts

1. Third step: Inspection

- Arms pressed against hips
- Pay attention to the normal condition of the breasts

1. Fourth step: Lying in the correct position

- Lying on back
- Placing a pillow or towel under the shoulder
- Placing one hand beneath the head

1. Fifth step: Breast palpation

- Placing three middle fingers of the opposite hand together
- Using finger pads
- Slow palpation of the breast surface in a circular pattern
- Palpation of the whole chest area
- Palpate around the breast area

1. Sixth step

- Squeezing the nipple noting for discharge

1. Palpation of lymph nodes

- Using finger pads to palpate both axillary areas
- Using finger pads to palpate superior and inferior part of clavicle
- Performing all mentioned steps in order of priority
